# Controllable Carrier Doping in Two-Dimensional Materials Using Electron-Beam Irradiation and Scalable Oxide Dielectrics

**DOI:** 10.3390/mi14112125

**Published:** 2023-11-19

**Authors:** Lu Wang, Zejing Guo, Qing Lan, Wenqing Song, Zhipeng Zhong, Kunlin Yang, Tuoyu Zhao, Hai Huang, Cheng Zhang, Wu Shi

**Affiliations:** 1 State Key Laboratory of Surface Physics, Institute for Nanoelectronic Devices and Quantum Computing, Fudan University, Shanghai 200433, China; luwang21@m.fudan.edu.cn (L.W.); zjguo23@m.fudan.edu.cn (Z.G.); 22110190025@m.fudan.edu.cn (Q.L.); wqsong22@m.fudan.edu.cn (W.S.); 21210190036@m.fudan.edu.cn (K.Y.); 23110190097@m.fudan.edu.cn (T.Z.); zhangcheng@fudan.edu.cn (C.Z.); 2 Zhangjiang Fudan International Innovation Center, Fudan University, Shanghai 201210, China; 3 Shanghai Frontiers Science Research Base of Intelligent Optoelectronic and Perception, Institute of Optoelectronic and Department of Material Science, Fudan University, Shanghai 200433, China; 23110300050@m.fudan.edu.cn (Z.Z.); huangh@fudan.edu.cn (H.H.)

**Keywords:** 2D materials, field-effect transistors, oxide dielectric, electron-beam doping, charge-trapping

## Abstract

Two-dimensional (2D) materials, characterized by their atomically thin nature and exceptional properties, hold significant promise for future nano-electronic applications. The precise control of carrier density in these 2D materials is essential for enhancing performance and enabling complex device functionalities. In this study, we present an electron-beam (e-beam) doping approach to achieve controllable carrier doping effects in graphene and MoS_2_ field-effect transistors (FETs) by leveraging charge-trapping oxide dielectrics. By adding an atomic layer deposition (ALD)-grown Al_2_O_3_ dielectric layer on top of the SiO_2_/Si substrate, we demonstrate that controllable and reversible carrier doping effects can be effectively induced in graphene and MoS_2_ FETs through e-beam doping. This new device configuration establishes an oxide interface that enhances charge-trapping capabilities, enabling the effective induction of electron and hole doping beyond the SiO_2_ breakdown limit using high-energy e-beam irradiation. Importantly, these high doping effects exhibit non-volatility and robust stability in both vacuum and air environments for graphene FET devices. This methodology enhances carrier modulation capabilities in 2D materials and holds great potential for advancing the development of scalable 2D nano-devices.

## 1. Introduction

Field-effect transistors based on atomically thin 2D materials represent fundamental building blocks for next-generation electronic devices [1,2]. Traditional field-effect gating, which relies on solid dielectric layers, plays a pivotal role in enabling the transistor operation and consequently in realizing various device functions. However, conventional field-effect gating also faces inherent challenges, including the difficulty of achieving ultrahigh carrier concentration beyond the dielectric breakdown limit and precisely defining nanoscale doping profiles [3,4,5,6,7,8]. To address these challenges, researchers have been exploring alternative doping methods to enhance transistor performances beyond the capabilities of solid gates. For instance, ionic gating allows for ultrahigh carrier concentrations in transistors, but also introduces disorder and is unsuitable for local doping [9,10,11]; photoinduced doping can induce high carrier densities while maintaining device mobilities, but it suffers from limited spatial resolution in local doping [12,13,14,15]. 

In previous studies, we introduced a fully reversible e-beam doping technique that can realize controllable doping effects in graphene/h-BN and MoS_2_/h-BN heterostructures on SiO_2_/Si substrates [16,17]. This technique is capable of achieving ultrahigh carrier concentration beyond the dielectric breakdown limit while preserving high device mobilities. It also facilitates the creation of nanoscale local doping patterns, illustrating its potential for applications in creating nanoscale circuitry and prototyping of innovative nano-devices. This flexible e-beam doping technique is, thus, an ideal approach to expand the FET functionalities of various 2D materials. However, the e-beam doping technique is reliant on the dielectric layers within the device structure, as the e-beam-induced doping effects stem from charged defects in these dielectric layers [16,17,18,19,20,21,22]. In prior demonstrations, the use of h-BN as a dielectric layer was essential and played a pivotal role in achieving controllable doping [16,17]. Nevertheless, the scalability of h-BN as a dielectric layer is limited, which hinders its application in emerging large-area or array devices. Consequently, it is necessary to develop a scalable device structure that is more compatible with CMOS techniques to facilitate the implementation of this e-beam doping technique in scalable device applications. 

In this study, we replace the h-BN dielectric layer with an atomic layer deposition (ALD)-grown thin oxide layer of Al_2_O_3_ in graphene and MoS_2_ FET devices on SiO_2_/Si substrates. We demonstrate that controllable e-beam doping effects can be achieved in this scalable device structure. The ALD-grown Al_2_O_3_ layer forms an oxide interface with the SiO_2_ layer, effectively trapping charges activated by the high-energy e-beam irradiation and enabling both high electron and hole doping beyond the breakdown limit of the SiO_2_ dielectric. Furthermore, these substantial doping effects can be induced rapidly and exhibit non-volatile characteristics, with excellent stability in both vacuum and air environments for graphene FET devices. Our e-beam doping technique, combined with a device structure that incorporates scalable oxide layers, extends to other 2D materials such as MoS_2_, emphasizing its versatility and potential in broadening the functionalities of scalable 2D nano-devices. 

## 2. Device Structure and Methods

**Figure 1:**
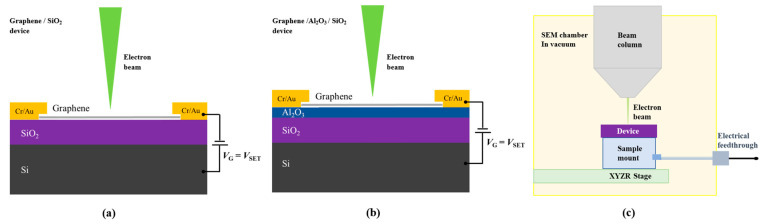
Schematic of electron-beam (e-beam) doping in graphene FET devices on SiO_2_/Si substrates (**a**) without and (**b**) with an ALD-grown Al_2_O_3_ layer. Charge doping is induced in the device during e-beam (1–30 keV) exposure in a standard SEM for a few seconds while holding the back-gate voltage *V*_G_ = *V*_SET_ ≠ 0 V. (**c**) Experimental setup for in situ e-beam doping and electrical measurements. The device is mounted in a SEM chamber using a custom holder attached to an electrical feedthrough for e-beam doping and in situ transport measurements.

In our experiments, we first compared the e-beam doping effects between two distinct FET device structures, as depicted in Figure 1a,b. Figure 1a presents the typical graphene FET device on a standard SiO_2_/Si substrate, while Figure 1b showcases an FET device featuring a thin oxide layer Al_2_O_3_ on top of the SiO_2_/Si substrate. The thin oxide layer Al_2_O_3_ was grown via ALD (model: MNT-S100-L4S1). Among the various growth techniques for fabricating Al_2_O_3_ films, ALD has its unique advantage of precise control over thickness and composition at atomic level, which is based on its sequential and self-limiting reaction [23]. The detailed process of ALD-grown Al_2_O_3_ is as follows: the SiO_2_/Si substrates were sequentially cleaned with acetone, isopropanol, and deionized water prior to deposition. Trimethylaluminum (TMA, Al(CH_3_)_3_), H_2_O, and Ar were used as the Al precursor, oxygen source, and purge gas, respectively. The Al_2_O_3_ film was grown at the temperature of 200 centigrade with a rate of 0.115 nm/cycle. The Al_2_O_3_ thin oxide layer grown on SiO_2_/Si substrates had a film thickness of 50 nm, controlled by the number of 435 ALD cycles. The ALD process sequence was: Al(CH_3_)_3_ (20 ms)–Ar purge (20 s)–H_2_O (20 ms)–Ar purge (25 s). This extra oxide layer serves to establish an interface between two dielectric materials, significantly enhancing the charge-trapping capabilities during the e-beam doping process. The ALD-grown oxide layer can be manufactured with a larger area, offering advantages over cleaved h-BN in terms of scalability for device applications. The mechanically exfoliated graphene or MoS_2_ flakes were transferred on top of standard SiO_2_/Si substrates with or without featuring thin oxide layer ALD-grown Al_2_O_3_.

Figure 1c provides a schematic diagram of the experimental setup for in situ e-beam doping and electrical transport measurements. We mounted the FET devices within a scanning electron microscope (SEM) (model: TESCAN Vega) and conducted in situ measurements through a high vacuum multiterminal electrical feedthrough, installed on a modified SEM port. During e-beam irradiation, an AC bias is applied to the sample (50 μV for graphene devices and 50 mV for MoS_2_ devices in this study) to monitor channel conductance. A gate voltage, *V*_G_, relative to the source, is applied to modulate the carrier density of the channel during exposure. Channel conductance is measured using a lock-in amplifier (SR830, Stanford Research Systems, Sunnyvale, CA, USA), while the gate voltage/current is sourced/measured through a Keithley source meter (Keithley 2400 or 2450, Tektronix, Beaverton, OR, USA). All electrical measurements were conducted at room temperature. 

We utilized electron-beam energies ranging from 1 keV to 30 keV, with a beam current (*I*_e_) varying from 1 pA to 15 pA for e-beam-induced doping. In this work, the normal scanning mode was employed to investigate the doping effects on the graphene and MoS_2_ FET devices. The lithography mode of the SEM, although not explicitly illustrated in this context, can be utilized to realize multiple functions, such as the direct inscription of local doping patterns onto the 2D FET devices. The exposure conditions for e-beam doping remained consistent with those used in prior studies [16,17]. In the normal scanning mode, the typical exposed area (*S*) was approximately 5000 μm^2^, encompassing the entire sample region. The typical exposure time (*t*) ranged from 5 to 120 s. The cumulative irradiation dosage (*D*) can be calculated as *D* = *I*_e_*t*/*Se*, where *e* is the elementary charge. The resistance was monitored during e-beam exposure with a preset gate voltage (*V*_SET_), and the exposure stopped when the irradiation-induced resistance change had stabilized. The overall doping effects were characterized by measuring the transfer curves of the FET devices after each e-beam doping process.

## 3. Results and Discussion

### 3.1. E-Beam Doping Effects in Graphene FET Devices on a Standard SiO_2_/Si Substrate

**Figure 2:**
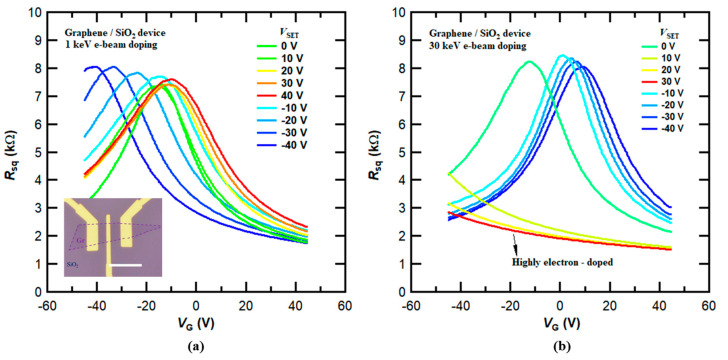
Transport characteristics of a graphene FET device on SiO_2_/Si substrate after e-beam-induced charge doping at 1 keV and 30 keV. (**a**) Sheet resistance *R*_sq_ (*V*_G_) of graphene with multiple e-beam-induced doping at a beam energy of 1 keV. The curves are obtained after e-beam doping with *V*_G_ = *V*_SET_ ranging from −40 V to 40 V (from blue to red). Inset: optical image of the device. Scale bar, 10 μm. (**b**) Same as in (**a**), but at a beam energy of 30 keV. The device is highly electron-doped after 30 keV e-beam doping with a preset gate voltage *V*_SET_ = 30 V.

**Figure 3:**
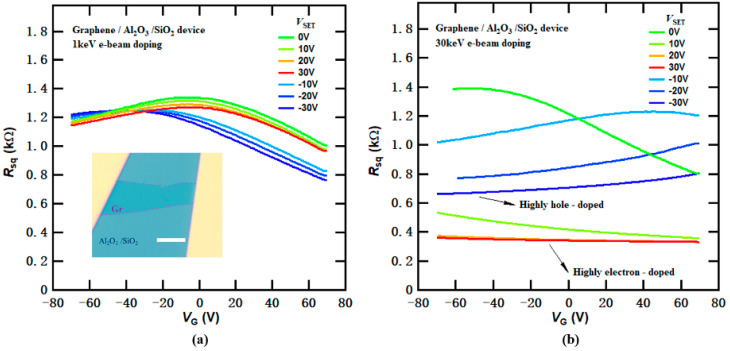
Transport characteristics of a graphene FET device with an ALD-grown Al_2_O_3_ layer on top of SiO_2_/Si substrate after e-beam-induced charge doping at 1 keV and 30 keV. (**a**) Sheet resistance *R*_sq_ (*V*_G_) of graphene with multiple e-beam-induced doping at a beam energy of 1 keV. The curves are obtained after e-beam doping with *V*_G_ = *V*_SET_ ranging from −30 V to 30 V (from blue to red). Inset: optical image of the device. The thickness of the Al_2_O_3_ layer is 50 nm. Scale bar, 10 μm. (**b**) Same as in (**a**), but at a beam energy of 30 keV. The device is highly electron (hole)-doped after 30 keV e-beam doping with *V*_G_ = *V*_SET_ = 30 V (−30 V).

We first investigated the e-beam doping effects in graphene devices without and with an ALD-grown 50 nm thick Al_2_O_3_ dielectric layer on SiO_2_/Si substrates. The optical images in the insets of Figure 2a and Figure 3a display monolayer graphene samples with the corresponding device structures depicted in Figure 1a,b. Following procedures similar to those demonstrated in previous studies [16,17], we applied various *V*_SET_ values during the e-beam irradiation process, leading to varying levels of doping within the same device. Figure 2a,b present the transport characteristics of the graphene device on a standard SiO_2_/Si substrate following successive doping with 1 keV and 30 keV e-beams at different *V*_SET_ settings ranging from −40 V to 40 V. We can see that the graphene device predominantly exhibits electron doping regardless of the *V*_SET_ value and beam energy. Furthermore, high-energy e-beams (30 keV), coupled with a large preset voltage, could induce highly electron-doped states within the device, as shown in the red curve in Figure 2b. Conversely, achieving highly hole-doped states remains unattainable. However, when the Al_2_O_3_ layer was integrated into the device structure, as exemplified in Figure 1b, it became feasible to induce both highly electron-doped and hole-doped states in graphene devices.

### 3.2. E-Beam Doping Effects in Graphene FET Devices with Additional Al_2_O_3_ Dielectric Layer

Figure 3a,b illustrate the transfer curves of the graphene device with Al_2_O_3_/SiO_2_ dielectrics after successive doping with 1 keV and 30 keV e-beams at various *V*_SET_ values. At low beam energies (1 keV), the e-beam doping effects can be controlled by *V*_SET_, although the overall doping effectiveness was relatively modest, as shown in Figure 3a. However, at higher beam energies (30 keV), both highly electron-doped and hole-doped states could be realized, even with relatively small *V*_SET_ values, as shown in Figure 3b. Compared to the results in Figure 2b, the graphene device reached substantially higher conducting states, with the charge neutrality point (CNP) extending well beyond the gate sweeping range of −70 V to 70 V. The maximum change in CNP positions far exceeds 140 V, highlighting the remarkable doping capability beyond the SiO_2_ dielectric breakdown limit. These results clearly demonstrate that the doping effects rely on device structures and the additional Al_2_O_3_ layer plays a critical role in achieving high levels of doping. 

However, we have to note that the graphene device featuring Al_2_O_3_/SiO_2_ dielectrics exhibits somewhat reduced carrier mobility compared to the typical graphene FET device on a standard SiO_2_/Si substrate. This reduction could be attributed to Coulomb scattering from surface-trapped states or charged point defects (e.g., oxygen vacancies) from the ALD-grown oxide layer of Al_2_O_3_ [24,25]. As reported in previous studies, e-beam irradiations can induce lattice defects and vacancies in graphene and other 2D materials [18,21,22,26], thereby reducing the carrier mobility, especially under exposure to a large dosage or high-energy e-beam irradiation. For instance, Childres et al. observed a significant mobility decrease under 30 keV e-beam irradiation with a large dosage of 4500 e^−1^/nm^2^, while the mobility was preserved with a much smaller dosage of 112.5 e^−1^/nm^2^ for a graphene device on a SiO_2_/Si substrate [18]. In contrast, in our work, the maximum dosage irradiated on the graphene device is approximately 2.25 e^−1^/nm^2^, significantly lower than the dosage in previous reports [18]. Such a minimal dosage of irradiation should result in a negligible adverse impact on carrier mobilities. Additionally, the e-beam doping process on graphene FET devices is fully reversible and reproducible, indicating that permanent damages such as lattice defects or vacancies are unlikely to appear in the graphene devices under e-beam irradiation in our experiments.

**Figure 4:**
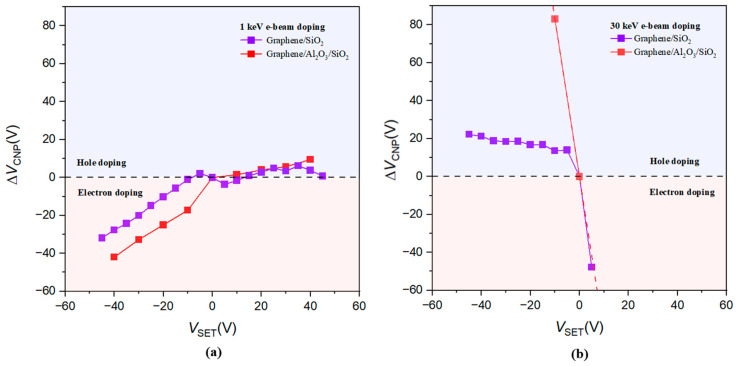
Summary of e-beam-induced doping effect at 1 keV and 30 keV in graphene FET devices. (**a**) The CNP shift *ΔV*_CNP_ (proportional to the doped carrier concentration) versus the corresponding *V*_SET_ for the e-beam doping at 1 keV. Purple and red curves represent results measured from graphene FET devices on SiO_2_/Si substrates without and with an ALD-grown Al_2_O_3_ layer, respectively. Red and blue shaded regions indicate electron and hole doping induced by e-beam exposure, respectively. (**b**) Same as in (**a**), but at a beam energy of 30 keV.

We summarize the e-beam doping effects by plotting the shift of the CNP position, denoted as Δ*V*_CNP_ (relative to the doped state with *V*_SET_ at 0 V), as a function of the preset voltage *V*_SET_. These results are shown in Figure 4a,b. As shown in Figure 4a, the observed electron and hole asymmetric doping behavior in the graphene device on the standard SiO_2_/Si substrate can be attributed to the preferential trapping of holes, as opposed to electrons, in the SiO_2_ layer under electron beam irradiation [27]. Previous research has indicated that electrons possess significantly higher mobility than holes, and that hole traps are more abundant than electron traps in SiO_2_ [28]. However, with the presence of an additional Al_2_O_3_/SiO_2_ interface, as illustrated in Figure 1b, electrons and holes generated in Al_2_O_3_ or the SiO_2_ layer can become trapped near the interface. This can lead to either n-type or p-type high doping in the graphene device, as displayed in Figure 4b. 

**Figure 5:**
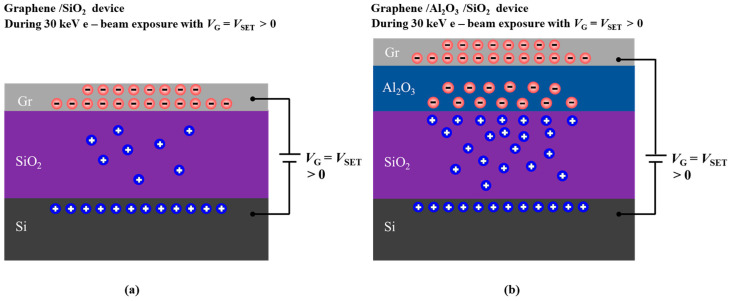
Proposed mechanism for the electron-beam-induced doping effect at 30 keV in graphene FET devices. (**a**) Schematic of the doping process and charge carrier distribution for 30 keV e-beam doping in graphene FET devices on SiO_2_/Si substrates for 30 keV e-beam doping at a positive preset voltage *V*_SET_. (**b**) Schematic of the doping process and charge carrier distribution for 30 keV e-beam doping in graphene FET devices on Al_2_O_3_/SiO_2_/Si substrates for 30 keV e-beam doping at a positive preset voltage *V*_SET_.

In Figure 5, we provide a simplified model of the charge distribution during the e-beam doping process in the graphene devices to illustrate the potential doping mechanism. During irradiation of the graphene devices, there are a variety of processes occurring (secondary electron emission, plasmon decay, etc.) [29], but the primary effects that contribute to the doping are electron–hole pair generation and trap states within the dielectrics [28]. In Figure 5a, electron–hole pairs are generated in SiO_2_ under e-beam irradiation. Some of these pairs separate due to the electric field (*E*) induced by the gate voltage (*V*_SET_) applied between the graphene and the degenerately doped silicon. However, due to the unstable trap states, electron–hole pairs tend to recombine again. Consequently, SiO_2_ reverts to its initial state of preferentially trapping holes. This leads predominantly to the electron doping of the graphene, regardless of *V*_SET_. 

When the Al_2_O_3_ layer is present, as shown in Figure 5b, this electric field (*E*) causes electron–hole pairs in the Al_2_O_3_ and SiO_2_ layers to separate and drift in opposite directions. Electrons and holes then move towards the Al_2_O_3_-SiO_2_ interface and become trapped in defect states near the interface without recombination. Here, by modulating the direction of the electric field with positive and negative *V*_SET_ values, the carrier type of charged trap states near the Al_2_O_3_-SiO_2_ interface can be switched between the electrons and holes. Consequently, graphene can be both effectively electron- and hole-doped, even when the gate is disconnected. In the Al_2_O_3_/SiO_2_ double-oxide device configuration, the Al_2_O_3_ dielectric layer serves a function similar to h-BN in previous studies [16,17], suggesting a similar potential mechanism. 

### 3.3. E-Beam Doping Effects in MoS_2_ FET Devices with Additional Al_2_O_3_ Dielectric Layer

**Figure 6:**
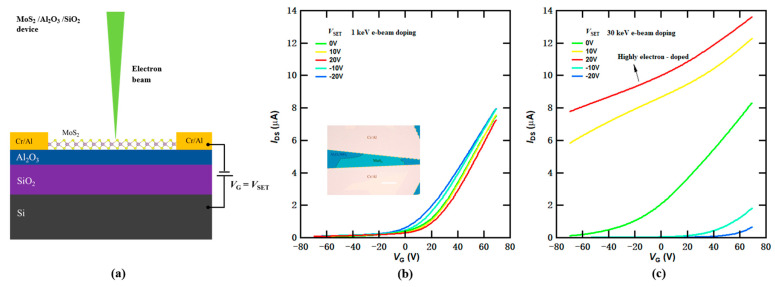
Electron-beam-induced doping effect in a MoS_2_ FET device with an ALD-grown Al_2_O_3_ layer on top of SiO_2_/Si substrate. (**a**) Schematic of e-beam doping in the MoS_2_ FET device on Al_2_O_3_/SiO_2_/Si substrate. (**b**,**c**): Transfer curves *I*_DS_ (*V*_G_) of MoS_2_ after e-beam doping with *V*_G_ = *V*_SET_ ranging from −20 V to 20 V (from blue to red) at 1 keV and 30 keV, respectively. The device is highly electron-doped with the threshold voltage well beyond −80 V after 30 keV e-beam doping at a preset voltage *V*_SET_ = 20 V. With e-beam doping at *V*_SET_ = −20 V, the threshold voltage is substantially shifted to the right, indicating an opposite doping effect, as shown in (**c**). Inset in (**b**): optical image of the device. The thickness of the Al_2_O_3_ layer is 40 nm. Scale bar, 20 μm.

The e-beam doping process, in conjunction with the double-oxide device geometry, is also applicable to various other 2D materials. We fabricated MoS_2_ FET devices featuring an ALD-grown 40 nm thick Al_2_O_3_ dielectric layer on SiO_2_/Si substrates, as depicted in Figure 6a. By adopting a similar device structure that incorporates oxide interfaces, we successfully achieved controlled e-beam doping effects in MoS_2_ samples. Figure 6b,c show the transfer curves of the same MoS_2_ sample after successive e-beam doping with varying *V*_SET_ settings, using 1 keV and 30 keV e-beams, respectively. It is evident that the doping effectiveness remains limited at low beam energies, as displayed in Figure 6b. However, with high beam energies, highly doped states can be readily attained. As shown in Figure 6c, with a preset voltage of 20 V and exposure to a 30 keV e-beam, the MoS_2_ FET device turns into a heavily electron-doped state, resulting in a significant shift of the threshold voltage well beyond −80 V (as indicated by the red curve). Conversely, the threshold voltage is substantially shifted to the right (as shown in the blue curve) with a preset voltage of −20 V under 30 keV e-beam irradiation, indicating an opposite doping effect, causing electron depletion or hole doping. Since the MoS_2_ sample is originally electron-doped and has a substantial bandgap, hole conduction was not observed in the current device structure. Further optimization of the doping parameters and the device structure may be necessary to reach a highly hole-doped regime.

Moreover, considering that the devices in this work are directly exposed to electron beams, the direct impact of the electron beam on the semiconducting channel materials requires further investigation. An earlier report claimed that e-beam irradiation with a high energy of about 80 keV could create point defects and vacancies in MoS_2_, leading to a reduction in carrier mobilities [26]. In contrast, the maximum e-beam energy we employed is only 30 keV, which is significantly lower, and our exposure dosage remained minimal. Therefore, the proposed e-beam doping mechanism associated with charge trapping in oxide dielectrics should predominantly govern the overall doping effects in our experiments rather than the direct impact from e-beam irradiation. Furthermore, we can optimize the device configuration by adding a thin capping layer on top of the channel material, which can protect the device from e-beam irradiation-induced defects or vacancies, as demonstrated in our previous work with h-BN capping layers [16].

### 3.4. Response Time, Stability, and Repeatability of E-Beam Doping Effects

**Figure 7:**
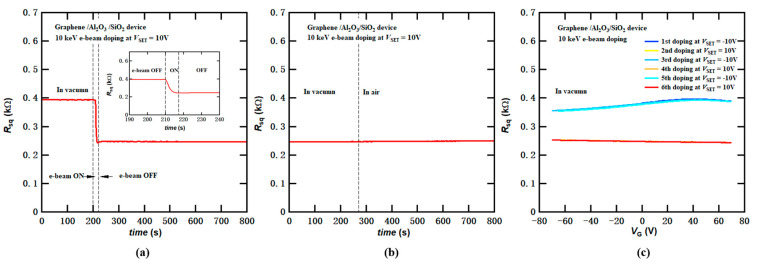
Response time, stability, and repeatability of graphene FET device on Al_2_O_3_/SiO_2_/Si substrate after e-beam-induced charge doping at 10 keV. (**a**) Sheet resistance change of graphene during e-beam doping process at a beam energy of 10 keV while maintaining *V*_SET_ at 10 V. The electron beam is unblanked at 210 s and blanked again at 217 s. Inset: a zoomed-in figure that demonstrates a short doping response time of only a few seconds. (**b**) Stability of e-beam-induced doping in the graphene FET device. Sheet resistance of graphene versus time after doping as the SEM chamber transitions from vacuum to an ambient environment. (**c**) Repeatability of e-beam-induced doping in the graphene FET device. *V*_SET_ alternates between 10 V and −10 V three times.

The e-beam-induced doping process exhibits a relatively fast response time. In Figure 7a, we present the resistance change measured in another graphene device with the device structure depicted in Figure 1b during the e-beam doping at an e-beam energy of 10 keV while holding *V*_SET_ at 10 V. The resistance rapidly decreases upon the unblanking of the e-beam, and stabilizes within a few seconds (see the inset in Figure 7a). This response time significantly outperforms that of photoinduced doping [12], which typically requires several minutes to manifest. 

The doped state remains stable even after the removal of both the e-beam and back-gate voltage, even under ambient conditions. As shown in Figure 7b, the resistance of the graphene device remained unchanged after 10 keV e-beam doping with *V*_SET_ set to 10 V, even when venting the SEM chamber to transit from a vacuum environment to ambient conditions. This suggests that the e-beam-induced doping effects exhibit non-volatile doping characteristics and excellent stability. It is worth noting that over an extended period, the doped sample may exhibit slow decay due to the influence of moisture and oxygen in the air. Additionally, if not properly controlled, exposure to light may induce additional photoinduced doping [12,13,14,15].

We also conducted tests to assess the repeatability of the e-beam doping process within the same graphene device. Initially, the same graphene device was doped under 10 keV e-beam irradiation with *V*_SET_ set to −10 V, and then it was re-doped with *V*_SET_ at 10 V. This doping cycle was repeated three times, and transfer curves were recorded after each doping process. The results, plotted in Figure 7c, reveal that the transfer curves remained consistent across multiple doping cycles, demonstrating the excellent repeatability of the e-beam doping technique. 

## 4. Conclusions

In this study, we successfully demonstrated the controllable and reversible induction of both high electron and hole doping in graphene and MoS_2_ FET devices through the utilization of the e-beam doping technique, in conjunction with the incorporation of a thin Al_2_O_3_ dielectric layer into the device structure. This new device geometry establishes an oxide interface, thereby enhancing charge-trapping capabilities compared to conventional single SiO_2_ devices. Therefore, effective control over electron and hole doping can be achieved through high-energy e-beam irradiation, coupled with adjustments to preset voltages. Importantly, the e-beam-induced high doping effects exhibit non-volatility and robust stability, both in vacuum and air environments, for graphene FET devices. Moreover, this e-beam doping approach with a double-oxide device geometry is readily applicable to various 2D materials, including MoS_2_. The versatility of this technique should extend to the attainment of similar doping effects using other scalable oxide dielectrics, such as ALD-grown HfO_2_. The careful choice of proper oxide dielectrics with different defect densities or dielectric constants may further enhance the doping capabilities and help reach an even higher electron or hole density regime. On the other hand, large areas of chemical vapor deposition (CVD)-grown 2D materials can be readily used as the FET channel materials to further scale the device functionalities.

Another significant advantage of the e-beam doping technique lies in its capacity to facilitate high-resolution local doping pattern creation through the SEM’s lithography function. In previous studies [16], high-quality P-N junctions and pre-designed local doping patterns with a 200 nm line width have been demonstrated using the lithography mode of e-beam exposure. Such capability should be well maintained with the new device configuration. Thus, our work introduces a new methodology with the potential to advance the development of scalable nano-circuitry and facilitate the rapid prototyping of functional nano-devices. 

## Data Availability

The data are available from the corresponding author upon reasonable request.
